# Germ Cell Neoplasia in Situ Recognized Incidentally with Complaining of Discomfort in the Right Testis: A Case Report

**DOI:** 10.14789/jmj.JMJ23-0016-CR

**Published:** 2023-08-25

**Authors:** KEIJI TAKAHASHI, KAZUHIKO MIZUSHIMA, MAI YAMAZAKI, NAOKO TAKAZAWA, HIDEYUKI ISOBE, MIKI ASAHINA, SHU HIRAI, SHIGEO HORIE

**Affiliations:** 1 Department of Urology, Juntendo University, Graduate School of Medicine, Tokyo, Japan; 1 Department of Urology, Juntendo University, Graduate School of Medicine, Tokyo, Japan; 2 Department of Diagnostic Pathology, Juntendo Tokyo Koto Geriatric Medical Center, Juntendo University, Tokyo, Japan; 2 Department of Diagnostic Pathology, Juntendo Tokyo Koto Geriatric Medical Center, Juntendo University, Tokyo, Japan; 3 Department of Urology, Juntendo Tokyo Koto Geriatric Medical Center, Juntendo University, Tokyo, Japan; 3 Department of Urology, Juntendo Tokyo Koto Geriatric Medical Center, Juntendo University, Tokyo, Japan

**Keywords:** testicular cancer, germ cell neoplasia in situ, testis

## Abstract

A 27-year-old man experienced discomfort in his right testis in early September, 2021, and visited the hospital five days later. Physical examination did not detect any abnormalities in the scrotum. However, an ultrasound revealed a tumor in the central part of the right testis, and a Magnetic Resonance Imaging (MRI) showed a tumor 2.7cm in diameter with clear boundaries and a marginally smooth surface.

The level of alpha-fetoprotein, human chorionic gonadotropin, human chorionic gonadotropin-β subunit, and lactate dehydrogenase were within normal limits. A Computed Tomography (CT) scan showed no abnormalities. We can't rule out the possibility of malignancy, right radical orchiectomy was performed with a diagnosis of right testicular tumor in mid-September 2021.

The macroscopic lesion was 1.5×1.3 cm in size, and no viable tumorous cells were found pathologically. Atypical cells were observed in the seminiferous tubules from the spermatic cord, which were positively stained with immune-histochemical staining CD117 (c-kit), D2-40, and MIB-1 but negatively with alpha-fetoprotein, human chorionic gonadotropin, and human chorionic gonadotropin-β subunit.

The pathological diagnosis was germ cell neoplasia in situ, and no continuity was observed between these cells and bleeding necrosis.

The patient has been followed up for 1 year and 4 months after surgery, with no recurrence or metastasis observed.

## Introduction

Recently, germ cell neoplasia in situ (GCNIS) has been well recognized as a precursor lesion to testicular germ cell tumor. Although GCNIS is frequently found in association with germ cell tumors such as seminoma, isolated reports of GCNIS are relatively rare in the literature. In this report, we present a case of GCNIS incidentally discovered in a patient who presented with discomfort in the right testis. We also provide a review of previously reported cases of GCNIS.

## Case report

**Figure 1:**
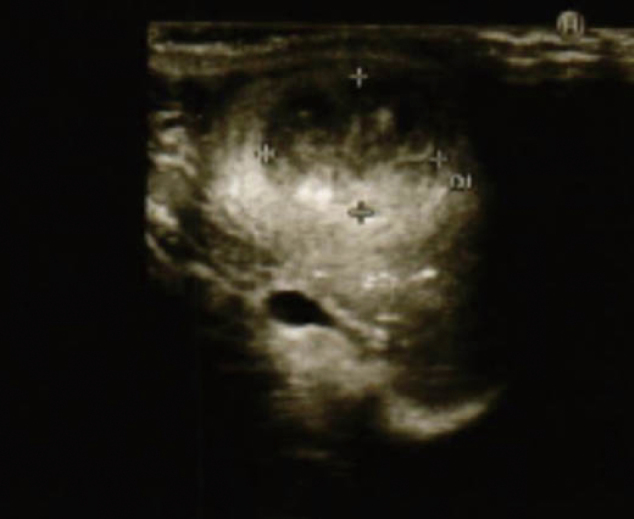
Ultrasound reveals heterogeneous neoplastic lesion in the right testis

**Figure 2:**
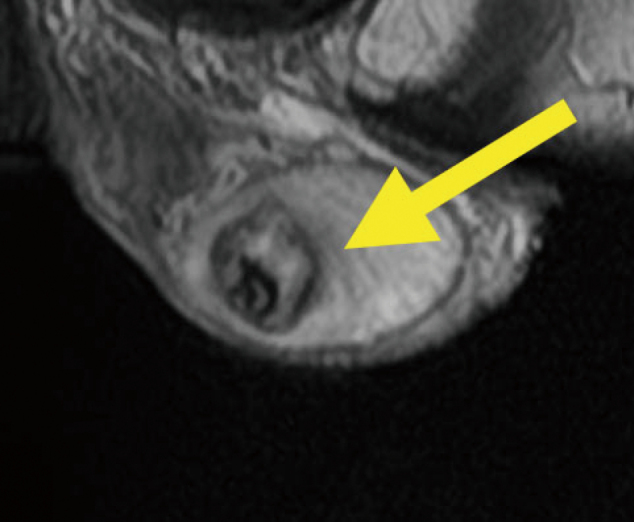
MRI reveals 2.7cm in diameter, clear boundary, and marginally smooth tumor in the right testis

**Figure 3:**
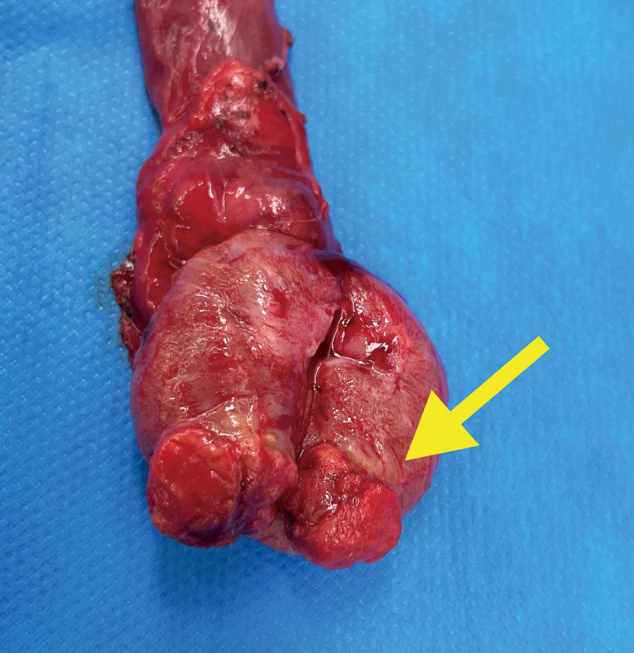
A solid mass 1.5cm in diameter in the right testis

A 27-year-old man presented with discomfort in the right testis in early September, 2021 and visited our department shortly thereafter. No abnormalities were found on palpation of the scrotum. Ultrasound revealed heterogeneous neoplastic lesion in the central part of the right testis (Figure 1). MRI revealed 2.7cm clear boundary and marginally smooth tumor in the center of the testis (Figure 2). Alpha-fetoprotein level was 1.38 ng/ml, human chorionic gonadotropin level was less than 1.0 mIU/l, human chorionic gonadotropin-*β* subunit level was less than 0.1ng/ml, and lactate dehydrogenase level was 178 U/l. CT revealed no abnormalities. We can't rule out the possibility of malignancy, right radical orchiectomy was performed with a diagnosis of right testicular tumor in mid-September 2021. The cut surface showed a solid mass 1.5cm in diameter in the right testis (Figure 3).

**Figure 4:**
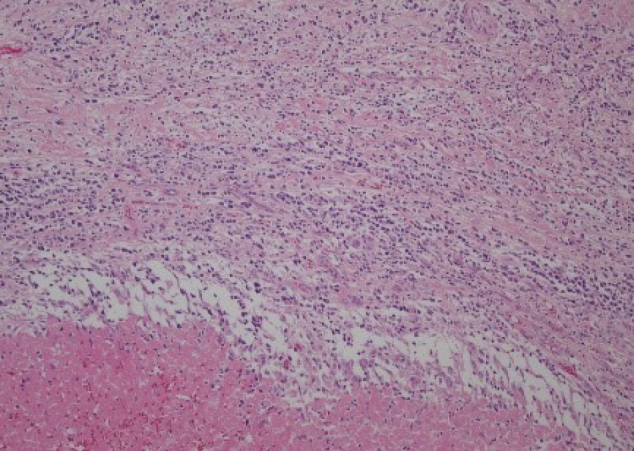
H&E staining × 100 Lower part is hemorrhagic necrosis area. No tumor was seen in the bordering granulation tissue.

**Figure 5:**
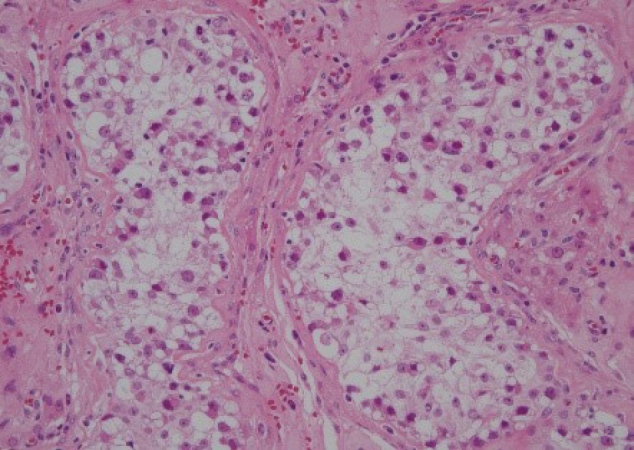
H&E staining × 200 Atypical cells do not extend beyond the basement membrane. Tumor cells are large, composed of a pale cytoplasm and large, irregular nuclei with well-defined nucleoli.

**Figure 6:**
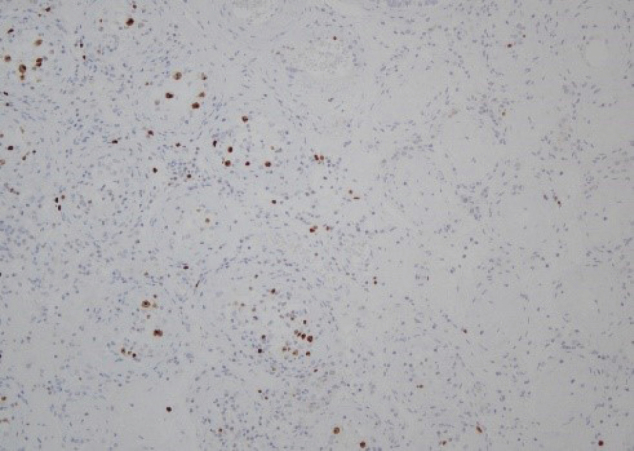
MIB-1 staining × 100 The tumor cell nuclei stain blackish brown color. 30-40% are positive, indicating cancer.

**Figure 7:**
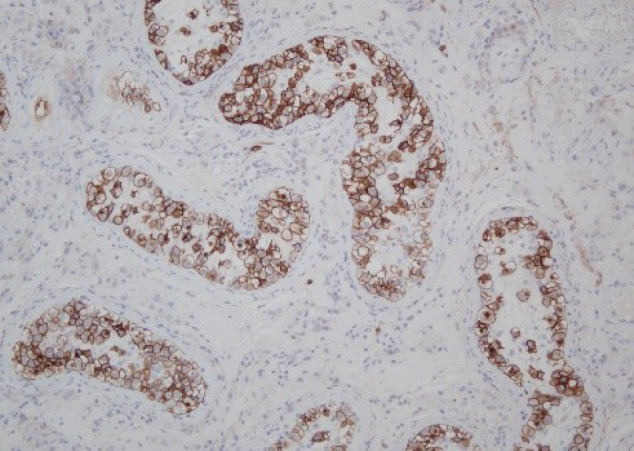
KIT (CD117) staining × 100 The tumor cell cytoplasm stains brown color. Consistent with GCNIS diagnosis.

**Figure 8:**
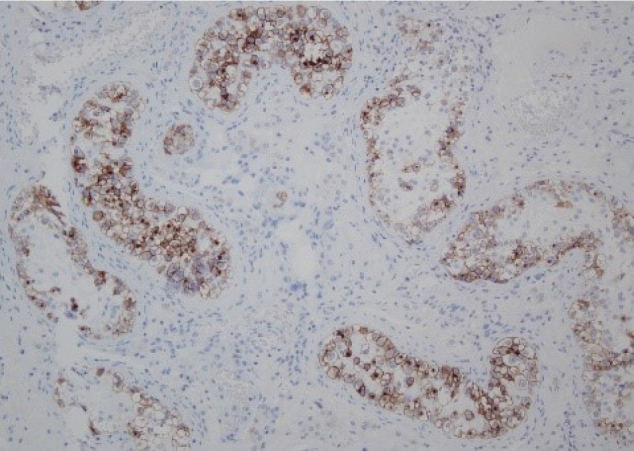
D2-40 (podoplanin) staining × 100 The tumor cell membrane stains brown color. Consistent with GCNIS diagnosis.

Bleeding necrosis and granulation tissue were observed in the tumorous lesion, but no viable tumorous cell component was observed pathologically. The granulation tissue intervened on the boundary line between necrosis and existing testicular tissue, but no findings suggestive of a tumor were obtained (Figure 4). The lesion contained atypical cells that were observed in the seminiferous tubules from the spermatic cord. The atypical cells were like spermatogonia, large, and had clear reticulum. The nucleus had large irregularity and included a clear nucleolus. These cells were arranged like lining the basement membrane. The collapse of the basement membrane or extravasation was not recognized. No extension to epididymis, vas deferens, and spermatic cord of atypical cells was seen (Figure 5). The surgical stump was negative for a tumor. The atypical cells were stained positively with immune-histochemical staining MIB-1 (Figure 6), CD117 (c-kit) (Figure 7) and D2-40 (Figure 8), but negatively with alpha-fetoprotein, human chorionic gonadotropin, and human chorionic gonadotropin-*β* subunit. No obvious continuity was observed between these cells and bleeding necrosis. From these results, the pathological diagnosis was germ cell neoplasia in situ (GCNIS). The patient's postoperative course was uneventful. After surgery, CT scans should be performed every 3 months according to the testicular tumor guidelines for the first year. Subsequently, CT scans should be conducted annually. He has no recurrence or metastasis 1 year and 4 months after surgery.

## Discussion

World Health Organization (WHO) revised its classification regarding urinary tract and male genital tumors in 2016. One significant change was observed with testicular germ cell tumors. Until now, the histopathological classification of testicular germ cell tumors was based only on morphological similarities. In contrast, the new classification prioritizes similarities in histogenesis over morphological similarities. Germ cell neoplasia in situ (GCNIS), previously known as intratubular germ cell neoplasia of unclassified type, is now considered to be a precursor of most testicular germ cell tumors except for spermatocytic tumors, yolk sac tumors, and mature teratomas.

According to the general rule for clinical and pathological studies on testicular tumors, the 2018 4th edition, GCNIS is regarded as a large tumor cell similar to germ cells that appear sparsely or in a row on basement membrane. GCNIS cells stain positively with immune-histochemical staining such as Placental alkaline phosphatase (PLAP), CD117(c kit), OCT3/4, SALL4, and D2-40. In the current case, large tumor cells were recognized as standing in a row, and those cells stained positively with immune-histochemical staining MIB-1, CD117 and D2-40, but negatively with alpha-fetoprotein, human chorionic gonadotropin, and human chorionic gonadotropin-*β* subunit.

As far as searched in Japan, there were only three cases where testicular germ cell tumors were not recognized other than biopsy, and the tumor was GCNIS alone^1)^. Testicular cancer occurs in 1% of men worldwide^2)^. On the other hand, CNIS is a precursor lesion to testicular germ cell tumors, and there is a report that 50% of them in progress to testicular cancer in 5 years and 70% of them in 7 years^3)^. There is a 1.9～5.2% chance that the testicular germ cell tumors occur contralaterally in heterochronous, and contralateral testicular biopsy during radical orchiectomy has been considered for the detection of GCNIS^4-6)^. The frequency of identification in contralateral testicular biopsy has been reported to be 3～5%^7)^.

It has been pointed out that the risk of contralateral onset of GCNIS is high in cases with low semen concentration, small testis volume, irregular internal echo image of the testis, and young patients^8)^. On the other hand, even if the result of contralateral testicular biopsy is negative, the development of tumors was about 1%, which can be considered a cause that the tumor was not detected due to low tumor burden^7, 9)^.

Two-part biopsy is considered to enhance the sensitivity of discovery. GCNIS was detected in 5.1% of prospective 2,318 case studies. The pathology of the biopsy specimens from the paired side was reported to be different in 31.1% of GCNIS-positive patients, and it was shown that the detection frequency increased with a two-part biopsy^10)^.

The complication rate of contralateral testicular biopsy includes hematoma and infection. The complication rate by two-part biopsy is reported to be less than 3%, and most complications resolved with conservative management, and the case requiring additional treatment was 0.6%^11)^. The treatment of GCNIS is radiation therapy, which can result in a complete cure. However, due to differences in the radiosensitivity of testicular cells, only Sertoli cells remain in the seminiferous tubules, causing azoospermia. As Leydig cells have low radiosensitivity, hormone replacement therapy is often not needed^12)^. It is reported that the an irradiation dose of 14 Gy causes 8% heterochronous incidence rate of germ cell tumor.

GCNIS will be 98% curable by irradiation dose of 18-20 Gy. But if the irradiation dose increases more than this, the possibility of hypogonadism will be increase.

Therefore, irradiation dose of 16-20 Gy is recommended for treatment^13)^. Although contralateral testicular biopsy is not recommended in Japan, it may be considered in high-risk patients groups.

Because testicular cancer primarily affects young people, cryopreservation of sperm is an important consideration if radiation therapy is chosen. In such case, providing enough information to the patient and obtaining informed consent is necessary.

As this case involves a young patient and there is some possibility of contralateral onset, careful longer-term follow-up is required^14)^.

## Conclusion

We described a case of GCNIS and reviewed the relevant literature. Since the patient is still young, longer-term follow-up is required.

## Funding

No funding was received.

## Author contributions

All authors read and approved the final manuscript.

## Conflicts of interest statement

The Author declares that there are no conflicts of interest.

## Informed consent

I obtained informed consent by providing an oral explanation of the study and receiving agreement from the participant.

## References

[1] Morikawa F, Sunagawa H, Takagi M, et al: A CASE OF INTRATUBLAR MALIGNANT GERM CELLS (ITMGC). Rinsho Hinyokika, 1996; 50: 971-973. (in Japanese)

[2] Huyghe E, Matsuda T, Thonneau P: Increasing incidence of testicular cancer worldwide: a review. J Urol, 2003; 170: 5-11. 10.1097/01.ju.0000053866.68623.da 12796635

[3] Rørth M, Rajpert-De Meyts E, Andersson L, et al: Carcinoma in situ in the Testis. Scand J Urol Nephrol Suppl, 2000; 166-186. 10.1080/00365590050509896 11144894

[4] Che M, Tamboli P, Ro JY, et al: Bilateral testicular germ cell tumors: twenty-year experience at M. D. Anderson Cancer Center. Cancer, 2002; 95: 1228-1233. 10.1002/cncr.10804 12216089

[5] Hemminki K, Liu H, Sundquist J: Second cancers after testicular cancer diagnosed after 1980 in Sweden. Ann Oncol, 2010; 21: 1546-1551. 10.1093/annonc/mdp562 20019089

[6] Colls BM, Harvey VJ, Skelton L, Thompson PI, Frampton CM: Bilateral germ cell testicular tumors in New Zealand: experience in Auckland and Christchurch 1978-1994. Oncol, 1996; 14: 2061-2065. 10.1200/JCO.1996.14.7.2061 8683237

[7] Kanto S, Hiramatsu M, Takeuchi A, et al: Carcinoma in situ detected by contralateral testicular biopsy of 55 germ cell tumor patients. Nihon Hinyokika Gakkai Zasshi, 2004; 95: 35-41. 10.5980/jpnjurol1989.95.35 14978939

[8] Rud CN, Daugaard G, Meyts ERD, et al: Sperm concentration, testicular volume and age predict risk of carcinoma in situ in contralateral testis of men with testicular germ cell cancer. J Urol, 2013; 190: 2074-2080. 10.1016/j.juro.2013.06.023 23770148

[9] Andreassen KE, Grotmol T, Cvancarova MS, Johannesen TB, Fossa SD: Risk of metachronous contralateral testicular germ cell tumors: a population-based study of 7, 102 Norwegian patients (1953-2007) Int J Cancer, 2011; 129: 2867-2874. 10.1002/ijc.25943 21626506

[10] Dieckmann KP, Kulejewski M, Pichlmeier U, Loy V: Diagnosis of contralateral testicular intraepithelial neoplasia (TIN) in patients with testicular germ cell cancer: systemic two-site biopsies are more sensitive than a single random biopsy. Eur Urol, 2007; 51: 175-183. 10.1016/j.eururo.2006.05.051 16814456

[11] Dieckmann KP, Heinemann V, Frey U, Pichlmeier U, German Testicular Cancer Study Group: How harmful is contralateral testicular biopsy? —an analysis of serial imaging studies and a prospective evaluation of surgical complications. Eur Urol, 2005; 48: 662-672. 10.1016/j.eururo.2005.06.008 16009484

[12] Dieckmann KP, Besserer A, Loy V: Low-dose radiation therapy for testicular intraepithelial neoplasia. J Cancer Res Chin Oncol, 1993; 119: 355-359. 10.1007/BF01208845 PMC12201197 8383689

[13] Kier MGG, Lauritsen J, Almstrup K, et al: Screening for carcinoma in situ in the contralateral testicle in patients with testicular cancer: a population-based study. Ann Oncol, 2015; 26: 737-742. 10.1093/annonc/mdu585 25542924

[14] The Japanese Urological association, The Japanese Society of Pathology, Japan Radiological Society, Japanese Society of Medical Oncology: General Rule for Clinical and Pathological Studies on Testicular Tumors (The 4th Edition). Tokyo: Kanehara Shuppan, 2018. (in Japanese)

